# Impact of empirical treatment in extended-spectrum beta-lactamase-producing Escherichia coli and Klebsiella spp. bacteremia. A multicentric cohort study

**DOI:** 10.1186/1471-2334-12-245

**Published:** 2012-10-05

**Authors:** Galo Peralta, María Lamelo, Patricia Álvarez-García, María Velasco, Alberto Delgado, Juan Pablo Horcajada, María Montero, María Pía Roiz, Maria Carmen Fariñas, Juan Alonso, Luis Martínez Martínez, Alfonso Gutiérrez-Macías, Jose Angel Alava, Azucena Rodríguez, Ana Fleites, Vicente Navarro, Elia Sirvent, Jose Antonio Capdevila

**Affiliations:** 1 Instituto de Formación e Investigación Marqués de Valdecilla (IFIMAV), 3ª Planta. Edificio IFIMAV. Avda Cardenal Herrera Oria s/n, 39011, Santander, Spain; 2 Hospital Montecelo, Pontevedra, Spain; 3 Hospital Universitario Fundación Alcorcón, Madrid, Spain; 4 Hospital del Mar, Barcelona, Spain; 5 Hospital Universitario Marqués de Valdecilla-IFIMAV, Santander, Spain; 6 Hospital de Basurto, Bilbao, Spain; 7 Hospital Central de Asturias, Oviedo, Spain; 8 Hospital de Torrevieja, Torrevieja, Spain; 9 Hospital de Mataró, Mataró, Spain

**Keywords:** Bacteremia, Extended-spectrum β-lactamase, Gram negative, Antibiotic empirical treatment, Prognosis

## Abstract

**Background:**

The objective of this study is to analyze the factors that are associated with the adequacy of empirical antibiotic therapy and its impact in mortality in a large cohort of patients with extended-spectrum β-lactamase (ESBL) - producing *Escherichia coli* and *Klebsiella* spp. bacteremia.

**Methods:**

Cases of ESBL producing Enterobacteriaceae (ESBL-E) bacteremia collected from 2003 through 2008 in 19 hospitals in Spain. Statistical analysis was performed using multivariate logistic regression.

**Results:**

We analyzed 387 cases ESBL-E bloodstream infections. The main sources of bacteremia were urinary tract (55.3%), biliary tract (12.7%), intra-abdominal (8.8%) and unknown origin (9.6%). Among all the 387 episodes, *E. coli* was isolated from blood cultures in 343 and in 45.71% the ESBL-E was multidrug resistant. Empirical antibiotic treatment was adequate in 48.8% of the cases and the in hospital mortality was 20.9%. In a multivariate analysis adequacy was a risk factor for death [adjusted OR (95% CI): 0.39 (0.31-0.97); P = 0.04], but not in patients without severe sepsis or shock. The class of antibiotic used empirically was not associated with prognosis in adequately treated patients.

**Conclusion:**

ESBL-E bacteremia has a relatively high mortality that is partly related with a low adequacy of empirical antibiotic treatment. In selected subgroups the relevance of the adequacy of empirical therapy is limited.

## Background

*Escherichia coli* and *Klebsiella* spp. are the most important causal agents of Gram negative bacteremia both in hospital and community acquired bloodstream infections [1,2]. These Enterobacteriaceae are increasingly worldwide resistant to antimicrobials [3,4]. One of the main causes for Enterobacteriaceae resistance is the presence of extended-spectrum beta-lactamases (ESBL), a family of plasmid-encoded enzymes that hydrolyse and cause resistance to most of the beta-lactam antibiotics, including penicillins, monobactams and most cephalosporins. Because ESBL-producing Enterobacteriaceae (ESBL-E) are also frequently resistant to other non-related beta-lactam antibiotics as quinolones, trimethoprim-sulfamethoxazole, and aminoglycosides, the recommended empirical antibiotic therapy when these infections are suspected are wide spectrum antimicrobials, essentially carbapenems [5].

The high prevalence of ESBL-harboring Enterobacteriaceae has resulted in increased use of carbapenems, which exhibit potent activity against many ESBL-harboring microorganisms. This in turn has led to an emergence and increase in resistance to carbapenems among Enterobacteriaceae [6].

In this epidemic hallmark of ESBL-E infections information about its optimal antimicrobial therapy is needed. In this sense, ESBL-E are often susceptible *in vitro* to some antimicrobials as beta-lactam/beta-lactam inhibitors, and some data support their potential role in the treatment of these infections although information about this subject in clinical practice is quite limited.

The aim of this study is to analyze the impact of adequacy of empirical antibiotic therapy in mortality in a large cohort of patients with ESBL-E bacteremia.

## Methods

### Study population

We studied cases of ESBL-E bacteremia diagnosed from January 2004 through December 2008 in 19 Spanish Hospitals, included in the Infectious Diseases Research Group of the Spanish Society of Internal Medicine. Cases were identified using the microbiology laboratory databases and reviewed by internal medicine/infectious diseases physicians.

### Data collection and definitions

Patients were identified retrospectively and reviewed using a standardized data collection sheet. Only the first episode of mono-microbial bacteremia of each patient was selected for the study. All charts were reviewed. A multi-institutional database was developed including the following variables: age, sex, origin of bacteremia, dates of hospital admission and discharge, comorbidities, antimicrobial therapy, surgical procedures, hospitalization in an intensive care unit, and in-hospital mortality.

Bacteremia was considered to have been nosocomially acquired if appearing 48 hours after admission and no evidence of infection was present on admission. Episodes were considered health care–associated according to the criteria of Friedman et al [7]. The source of bacteremia was determined by clinical assessment and appropriate cultures (urine, sputum…) when available. Renal failure was defined by a creatinine value >2.0 mg/dL. Neutropenia was defined as an absolute neutrophil count of <500 cells/mm3 at the onset of the bacteremia. Immunosuppression was defined as the presence of neutropenia or HIV infection (with CD4 count <350 cells/mm3), or immunosuppressive therapy. Co-morbidities were assessed by using the Charlson co-morbidity score [8]. Sepsis, severe sepsis and septic shock were defined according to the American College of Chest Physicians/Society of Critical Care Medicine Consensus Conference [9]. Empirical antimicrobial therapy was defined as adequate in terms of *in vitro* susceptibility of an organism isolated, and if antibiotic treatment was started within 24 hours after drawing blood cultures. The correction for definitive therapy was defined as that implemented after blood cultures results was obtained in patients with inadequate empirical therapy, and also was considered adequate or inadequate in terms of *in vitro* susceptibility of an organism isolated. Oxyimino-beta-lactams (cefuroxime, cefotaxime, ceftriaxone, ceftazidime and aztreonam) were considered to be inappropriate, regardless of the MIC, for the treatment of infections caused by ESBL-producing *Enterobacteriaceae*. Therapy with urinary antiseptics such as norfloxacin, fosfomycin, pipemidic acid or nalidixic acid was considered inadequate [10]. Organisms reported as intermediate in susceptibility to a particular antibiotic were classified as non-susceptible for this report. ESBL-E showing resistance to at least 1 representative of 2 other families of antibiotics (fluoroquinolones, beta-lactam/beta-lactam combinations, or aminoglycosides) were considered multidrug resistant (MDR). The dosage of the antibiotic was not taken into account when assessing adequacy. All study definitions were established before data analysis. The study was approved by the Regional Institutional Review Board of Cantabria.

### Microbiological studies

ESBL-E isolation and identification were performed following standard methods at each centre. ESBL production was screened at the same time by the double disc synergy method as a complementary test for automated systems, at each centre. Minimal inhibitory concentrations were interpreted according to Clinical and Laboratory Standards Institute guidelines [11].

### Statistical analysis

Based on the results of the literature a sample size calculation was performed with the openepi program ( http://www.openepi.com). A mortality of 20% was considered in the adequate empirical treatment group and of 35% in the inadequate treatment group. For a power of 80% and a level of significance of 0.05, an overall sample of 270 patients was required (50% with adequate empirical treatment).

Categorical data were compared by the *x*2 or Fisher’s exact tests. Quantitative data were compared by Student’s *t*-test or the Mann–Whitney *U*-test, as appropriate. All reported p values are two-sided and have not been adjusted for multiple testing. Variables with a p crude value <0.1 in the univariate analysis were candidates for multivariate analysis and possible interactions were evaluated. The analysis was performed with the stepwise logistic–regression model of the SPSS software package. All subgroup analysis was *a priori* defined.

## Results

### Clinical features, adequacy of empirical therapy and temporal trends

The study included 399 patients detected in the participating centres. Excluded from the study were 12 (3%) patients due to insufficient data for the analysis. Among the remaining 387 patients, in hospital mortality was 20.9%. *E. coli* was isolated from blood cultures in 343 (88.6%) cases and *K. pneumoniae* in 44 cases (11.4%). The mean ± standard deviation patient age was 70.3 ± 16 years. Three patients were neonates (one with bacteremia caused by *E. coli* and two by *K. pneumoniae*). Seventy-two patients (18.6%) had severe sepsis and fifty-three (13.7%) had septic shock as a manifestation of the ESBL-E bacteremia. Median Charlson index was 3 (range 0-12). Table 1 shows the characteristics of the patients. Percentage of non-susceptibility of ESBL-E to antimicrobial was: quinolones: 82.9%, amoxicillin-clavulanate: 40.3%, gentamicin: 27.1%, piperacillin-tazobactam: 16.8% and carbapenem: 0%. In 45.7% of the cases the ESBL-E was multidrug resistant. In 189 (48.8%) episodes of bacteremia, the empirical antibiotic treatment was inadequate according to the microbiological results. In 44 (11.4%) episodes the definitive antibiotic treatment was inadequate. Of these patients with definitive inadequate therapy 24 (54.5%) patients died in the first three days after blood cultures extraction.

**Table 1:** Characteristics of the patients with ESBL-E bacteremia

**Demographics**	
Men	270 (62.5)
Age (mean, range)	70.3, 0-96 y
Age > 65 y	280 (72.4)
Place of acquisition	
Community acquired	110 (28.4)
Health care associated bacteremia	137 (35.4)
Nosocomial	140 (36.2)
Origin of infection	
Urinary	215 (55.3)
Biliary	49 (12.7)
Unknown	37 (9.6)
Intra-abdominal	34 (8.8)
Pneumonia	28 (7.2)
Catheter	16 (4.1)
Other	8 (2.1)
Comorbidity	
Diabetes	112 (28.9)
Cancer	111 (28.7)
Ictus	70 (18.1)
Dementia	66 (17.1)
Immunosuppression	65 (16.8)
COPD	65 (17.1)
Chronic renal failure	68 (17.6)
Cardiac failure	56 (14.5)
Liver cirrhosis	44 (11.4)
Metastatic tumor	33 (8.5)
Charlson ≥3	209 (54)

Date are no. (%) of patients; COPD: chronic obstructive pulmonary disease.

Of the 387 episodes of bacteremia 84 (21.7%) were detected in the period 2004-2005, and 303 (78.3%) in the period 2006-2008. When we compared the characteristics of the patients between these two periods we detected significant differences proportion in the frequency of empirical antibiotic treatment with piperacillin-tazobactam (7.3% vs. 14.9%, p = 0.03). Respect to E-ESBL resistance pattern, a trend to decrease in susceptibility from the first to the second period to amoxicillin-clavulanate (66.7% vs. 57.8%, p = 0.08) and decrease to ciprofloxacin (25% vs. 14.9%, p = 0.02) was detected and an increase in the proportion of multiresistant infections (35.7% vs. 48.5%, p = 0.02). A trend to decrease in the proportion of patients with community acquired infections was also detected (35.7% vs 26.4%, p = 0.06).

### Risk factors for mortality

The results of the univariate analysis of the risk factors associated with in hospital mortality are reflected in Table 2. Variables associated with mortality in the multivariate analysis were: hospital acquisition [adjusted OR (95% CI): 3.41 (1.89-6.18); P < 0.001], presence of metastatic disease [adjusted OR (95% CI): 4.2 (1.78-9.95); P = 0.001], adequacy of empirical antibiotic therapy [adjusted OR (95% CI): 0.39 (0.31-0.97); P = 0.04], presence of severe sepsis or shock [adjusted OR (95% CI): 11.66 (6.28-21.65) P < 0.001].

**Table 2:** Factors associated with mortality in patients with *E. coli* or *Klebsiella* spp. bacteremia

	**Death**	**Survival**	**p**	**RR (95 % CI)**
	**(n = 81)**	**(n = 306)**		
Demographics				
Men	57 (70.4)	207 (61.8)	0.1	1.36 (0.89-2.09)
Age > 65 y	61 (75.3)	219 (71.6)	0.3	1.17 (0.74-1.83)
Place of acquisition				
Community acquired	13 (16)	97 (31.7)	0.003	0.41 (0.22-0.78)
Health care associated	25 (30.9)	112 (36.6)	0.2	0.82 (0.53-1.24)
Nosocomial	43 (53.1)	97 (31.7)	<0.001	2 (1.36-2.93)
Origin of infection				
Urinary	38 (46.9)	177 (57.8)	0.05	0.71 (0.48-1.04)
Biliary	8 (9.9)	41 (13.4)	0.26	0.76 (0.39-1.47)
Unknown	9 (11.1)	28 (9.2)	0.36	1.18 (0.64-2.16)
Intra-abdominal	11 (13.6)	23 (7.5)	0.07	1.63 (0.96-2.77)
Pneumonia	8 (9.9)	20 (6.5)	0.21	1.41 (0.75-2.61)
Catheter	3 (3.7)	13 (4.2)	0.56	0.89 (0.32-2.52)
Comorbidity				
Diabetes	21 (25.9)	91 (29.7)	0.3	0.86 (0.55-1.34)
Ictus	15 (18.5)	55 (18)	0.51	1.03 (0.63-1.69)
Dementia	16 (19.8)	50 (16.3)	0.28	1.2 (0.74-1.93)
COPD	15 (18.5)	50 (16.3)	0.38	1.12 (0.69-1.84)
Chronic renal failure	20 (24.7)	48 (15.7)	0.05	1.54 (1-2.37)
Cardiac failure	15 (18.5)	41 (13.4)	0.16	1.34 (0.83-2.18)
Liver cirrhosis	16 (19.8)	28 (9.2)	0.009	1.92 (1.23-3)
Cancer	33 (40.7)	78 (25.5)	0.006	1.71 (1.17-2.51)
Metastatic tumor	13 (16)	20 (6.5)	0.009	2.05 (1.28-3.3)
Immunosuppression	16 (19.8)	49 (16)	0.25	1.22 (0.76-1.97)
Charlson ≥3	60 (74.1)	149 (48.7)	<0.001	2.43 (1.54-3.84)
Microbiology				
*E. coli*	65 (80.2)	278 (90.8)	0.009	0.52 (0.33-0.82)
Multidrug resistant	45 (55.6)	132 (43.1)	0.03	1.48 (1-2.19)
Presentation				
Sepsis severe or shock	57 (70.4)	68 (22.5)	<0.001	4.9 (3.2-7.51)
Adequate empirical therapy	34 (42)	164 (53.6)	0.04	0.69 (0.47-1.02)
Adequate change for definitive therapy	29 (32.1)	109 (35.6)	0.33	0.88 (0.58-1.34)

Date are no. (%) of patients.

A multivariate analysis selecting patients without severe sepsis or shock showed that adequate empirical therapy was not associated with mortality in this group, but it was associated with mortality in patients with severe sepsis or shock (adjusted OR, 0.42; 95% CI, 0.19–0.92; P = 0.03).

### Antibiotic therapy and prognostic factors in adequately treated patients

The most frequent empirical antibiotic treatments in the entire cohort were carbapenem (18.1%), amoxicillin/clavulanate (17.6%), ciprofloxacin or levofloxacin (16%), ceftriaxone or cefotaxime (12.9%) and piperacillin/tazobactam (12.9%).

A stratified analysis of mortality considering adequacy with different empirical antibiotic treatments is reflected in Table 3. For the group of patients who received adequate empirical treatment with beta-lactam/beta-lactam inhibitor combinations or with carbapenem mortality was 13.1% (11 of 84) and 24.6% (17 of 69) respectively (p = 0.05).

**Table 3:** **Mortality among patients with *E. coli *or *Klebsiella *spp. bacteremia treated empirically with different antimicrobials depending on the adequacy**

	**Adequate**	**Inadequate**	**p**	**RR (95% CI)**
Beta-lactam/Beta-lactam inhibitor combinations (n = 117)	11 (13.1)	13 (39.4)	0.002	0.58 (0.37-0.91)
Amoxicillin/clavulanate (n = 67)	3 (7.5)	10 (37)	0.004	0.34 (0.12-0.92)
Piperacillin/tazobactam (n = 50)	8 (18.2)	3 (50)	0.11	0.79 (0.54-1.14)
Quinolones (n = 62)	0)	10 (20.4)	-	-
Carbapenem (n = 70)	18 (25.7)	-	-	-
Cefotaxime or ceftriaxone (n = 50)	-	7 (14)	-	-
Aminoglycoside monotherapy* (n = 22)	3 (23.1)	2 (22.2)	0.68	1.02 (0.45-2.31)
None antimicrobial (n = 25)	-	7 (28)	-	-
Overall (n = 387)	34 (17.2)	47 (24.9)	0.04	0.78 (0.59-1.03)

Date are no. (%) of patients. *Patients with only aminoglycoside as empirical antibiotic ESBL-E susceptible.

A multivariate analysis of risk factors for death of the patients treated with adequate empirical antibiotics was performed, including the next variables: urinary origin, nosocomial acquisition, presence of neoplasm, presence of metastatic disease, Charlson index score ≥3, bacteremia caused by *E. coli,* MDR ESBL-E, and presence of severe sepsis or shock. In this model the risk factors associated with death were: presence of neoplasm [adjusted OR (95% CI): 4.32 (1.81-10.31); P = 0.001], presence of sepsis severe or shock [adjusted OR (95% CI): 8.62 (3.48-21.34); P < 0.001], and nosocomial acquisition [adjusted OR (95% CI): 2.93 (1.21-7.11); P = 0.02].

## Discussion

ESBL-E are rising worldwide as a cause of bloodstream community acquired, health related or nosocomial acquired infections [2-6,10,12-17]. In this context information about optimal therapeutic options for these infections in daily practice and their repercussion on outcome is needed. Several studies about ESBL-E bloodstream infections have analyzed the adequacy of empirical antibiotic therapy, risk factors for mortality and relationship of adequacy and outcome, with dissimilar results [6,12-15]. Discordance among series is relevant and is probably related, at least in part, with the limited number of cases in most of the studies, differences in the selected population, and differences in definitions. The present study represents one of the largest series even communicated of patients with ESBL-E bacteremia. Our results corroborate the relatively high mortality of ESBL-E bloodstream infections and underscore the relevance of adequacy of antibiotic treatment on mortality in ESBL-E bacteremia. Our study also reveals a role for beta-lactam/beta-lactam inhibitor combinations in the treatment of ESBL-E infections.

A triangle of relationships among resistance, adequacy and mortality in bloodstream infections has previously emphasized as one of the potential causes for the higher mortality of infections caused by MDR agents, including ESBL-E [5,13]. The general assumption of the causal connections of this triangle is the basis for the recommendation of wide spectrum antibiotic as empirical therapy in patients at risk of MDR infections. In ESBL-E infections carbapenems are widely considered as the drugs of choice on the basis on study cohorts, most of them retrospective, which suggests that third generations cephalosporins, irrespectively of *in vitro* susceptibility, and quinolones, are less effective for the treatment of these infections than carbapenems [15-17]. *In vitro* tests that indicate an inoculum effect for beta-lactam/beta-lactam inhibitor combinations, cephalosporins, and to a lesser extent with the quinolones, also support this practice [18]. As community acquired, health related and hospital acquired ESBL-E bloodstream infections are increasing, a raise in the empirical use of carbapenems in patients with suspected bloodstream infections and risk factors for ESBL-E acquisition is expected. In this hallmark with clinicians forced to use the carbapenems as the first choice for empirical treatment of severe infections, a future rise of the resistances to carbapenems has been predicted [19,20].

The exact role of beta-lactam/beta-lactam inhibitor combinations in the treatment of ESBL-E infections is matter of discussion [5-12]. Comparative clinical trials are lacking, and some recent reports suggest that beta-lactam/beta-lactam inhibitor combinations are potentially useful in the treatment of *in vitro* susceptible isolates [21,23]. A potential role of beta-lactam/beta-lactam inhibitor combinations in patients with ESBL-E bloodstream infections is suggested by our data. In our study mortality is consistently lower in patients treated empirically with carbapenems, or beta-lactam/beta-lactam inhibitor combinations when ESBL-E are susceptible although there are many confounding factors that impede us concluding consistently a role of beta-lactam/beta-lactam inhibitor combinations in empirical therapy for patients with ESBL-E bacteremia.

Another question that merit mention is the possibility of adjusting of antibiotic therapy using beta-lactam/beta-lactam inhibitor combinations or even quinolones after evaluating the susceptibility pattern of the ESBL-E in a “de-escalating” strategy. Our data suggest that beta-lactam/beta-lactam inhibitor combinations are potentially useful for treating ESBL-E susceptible to these antimicrobials, and could be an option for de-escalating.

Previous case series have detected a high variability in the origin of bacteremia, and while in the older reports the most frequent focus was respiratory tract [13,17,19] in some of the more recent studies, as in the present one, the most frequent origin of ESBL-E bacteremia are urinary and biliary tract [15,16]. This fact can be related to the higher incidence of hospital-acquired cases (i.e. ventilator associated pneumonia) at the beginning of the 2000’, and to the more recent “epidemic” increase of health-related and community acquired E-ESBL infections [24]. In relation to empirical antibiotic therapy this is relevant because an effect of adequacy related with the origin of infection has been previously detected [25], and suggested by our data. Its cause is unknown, although the lower mortality of ESBL-E bacteremia of urinary origin could influence this effect. Also we have detected a threefold increase in the number of cases of ESBL-E bacteremia between 2004-2005 and 2006-2008. A similar increase has been detected in previous studies.

Our study had several limitations. The study is retrospective and the comparisons between groups might be biased. The absence of a centralized laboratory to characterize ESBL-E strains and the absence of MICs are also limitation. Although the number of cases with ESBL-E is high in relation with previous studies; some subgroups have small size what may not have allowed us to find significant differences, especially regarding in hospital mortality.

In view of the increasing use of empirical carbapenems due to the risk of ESBL-E infections, information from clinical prospective studies about optimal empirical therapy for ESBL-E severe infections is needed. However the feasibility of these studies is uncertain as they imply the recruitment of a large number of patients with suspected ESBL-E infections to allow the collection of enough cases of microbiology proved infections for analysis. Meanwhile equilibrium among efforts to optimize initial therapy and efforts to promote judicious use of antimicrobial should be outweighed, and the role beta-lactam/beta-lactam inhibitor combinations in ESBL-E infections should be considered in specific hallmarks, essentially in de-escalating.

## Conclusions

Our results corroborate the relatively high mortality of ESBL-E bloodstream infections and underscore the relevance of adequacy of antibiotic treatment on mortality in ESBL-E bacteremia. Our study also reveals a role for beta-lactam/beta-lactam inhibitor combinations in the treatment of ESBL-E infections.

## Abbreviations

ESBL: Extended-spectrum beta-lactamase; ESBL-E: Extended-spectrum β-lactamase producing Enterobacteriaceae; MDR: Multidrug resistant; COPD: Chronic obstructive pulmonary disease.

## Competing interests

The authors declare that they have no competing interests.

## Authors’ contributions

GP, MV, and JAC have been involved in the conception and design; GP, ML, PAG, AD, MM, MPR, JA, AGM, JAA, AR, AF, VNL, and ESQ in the acquisition of data; and GP, LMM, MCF and in data analysis and interpretation. GP took the lead in drafting the manuscript, all authors critically reviewed manuscripts drafts and approved the final version.

## Pre-publication history

The pre-publication history for this paper can be accessed here:

http://www.biomedcentral.com/1471-2334/12/245/prepub
